# Adversarial debiasing for age-equitable diabetes prediction: performance–fairness trade-offs and partition dependency in machine learning

**DOI:** 10.3389/fdgth.2026.1816806

**Published:** 2026-07-17

**Authors:** Vinod Kumar Yata, Sravanthi Jena, Meera Indracanti, Shivaprasad Chitta, Narasaiah Kolliputi

**Affiliations:** 1 Department of Biotechnology, School of Allied and Healthcare Sciences, Malla Reddy University, Hyderabad, Telangana, India; 2 Department of Medical Biotechnology, School of Allied and Healthcare Sciences, Malla Reddy University, Hyderabad, Telangana, India; 3 Tridata Technologies, Tampa, FL, United States; 4 Division of Allergy and Immunology, Department of Internal Medicine, USF Morsani College of Medicine, Tampa, FL, United States

**Keywords:** adversarial debiasing, age bias, algorithmic fairness, diabetes prediction, gradient reversal layer, healthcare machine learning, partition dependency, recall parity

## Abstract

**Background:**

Machine learning models used for diabetes risk prediction may encode age-related biases that reduce diagnostic accuracy for specific demographic groups. Adversarial debiasing with a gradient reversal layer (GRL) offers a theoretically principled approach to learning representations that are invariant to a protected attribute; however, its practical effectiveness under realistic conditions of subgroup imbalance in healthcare datasets has not been fully characterised.

**Research question:**

Does adversarial debiasing with a GRL improve age-equitable diabetes prediction, and how do its fairness effects vary across different data partitions?

**Methods:**

Adversarial debiasing was evaluated for age-bias mitigation in diabetes prediction using the publicly available Pima Indians Diabetes Database (*n* = 768). All eight dataset predictors were used; three age groups (<30, 30–50, and >50 years) were derived from age for fairness evaluation. An adversarial neural model with a gradient reversal layer was compared against a logistic regression baseline. Features were standardised using a scaler fitted on training data only. The train–test split was stratified by diabetes outcome. Overall performance metrics (accuracy, recall, ROC-AUC) and the recall parity gap across age groups were computed on a primary labelled test partition (*n* = 154); robustness was assessed across five independent random seeds (0–4).

**Results:**

On the primary test partition, the adversarial model improved recall for the smallest age group [>50 years: 0.5556 → 0.7778, +22.22 percentage points (pp)] while maintaining comparable overall discrimination (ROC-AUC: 0.7852 → 0.7896, +0.45 pp). However, the recall parity gap increased from 0.0996 to 0.2153 (+11.57 pp), reflecting a concurrent decline in recall for the <30-year group (−6.25 pp). Across five random seeds, the mean recall parity gap showed a modest mean reduction (0.3282 → 0.3033, −2.49 pp), but with high variability (SD > 0.27) exceeding the mean difference. The adversarial model reduced the fairness gap in three of five seeds, increased it in one, and produced no change in one.

**Conclusion:**

Adversarial debiasing can improve predictive recall for underrepresented demographic subgroups but does not guarantee consistent fairness improvements across data partitions, particularly when subgroup sample sizes are small. Multi-seed evaluation is essential for reliable fairness assessment; single train–test splits are insufficient.

## 1 Introduction

Diabetes mellitus affects over 500 million adults worldwide and is a leading cause of cardiovascular disease, renal failure, blindness, and premature mortality. Early identification of individuals at high risk enables timely lifestyle interventions and preventive treatment that can delay or prevent disease onset (1, 29, 30), reducing both individual suffering and systemic burden on healthcare services. Machine learning models trained on clinical laboratory values and demographic data have shown promise in diabetes risk prediction (2, 31), potentially supporting population-level screening programs and targeted prevention efforts.

However, recent research has documented that clinical prediction models can exhibit systematic biases across demographic groups, producing less accurate predictions for racial minorities, women, and patients at the extremes of the age distribution (3). Such algorithmic bias raises serious ethical and clinical concerns. Biased models may exacerbate existing health disparities by providing inferior care to already underserved populations (4, 33), undermine trust in AI-enabled healthcare (5, 33), and potentially conflict with principles of medical ethics and anti-discrimination legislation (6). In diabetes prediction specifically, age-related bias is a clinically significant concern because diabetes risk and clinical presentation vary substantially across the lifespan (1), and older adults, who face the highest rates of complications, may be systematically underserved by models optimised for younger or middle-aged populations.

Algorithmic fairness in healthcare has become a recognised research priority (3, 32), with multiple technical approaches proposed to mitigate bias. Preprocessing methods rebalance training data; in-processing methods incorporate fairness constraints during model optimisation; and postprocessing methods adjust decision thresholds to equalise outcomes across groups. Among in-processing approaches, adversarial debiasing has attracted attention because of its theoretical elegance. By training a model to simultaneously predict the clinical outcome while failing to predict a protected attribute (e.g., age group), adversarial methods encourage the learning of representations invariant to sensitive attributes. This is typically implemented via a gradient reversal layer (GRL) that forces a shared representation to be uninformative about group membership while remaining predictive of the clinical target (7, 8).

Despite its theoretical appeal and encouraging results in computer vision and natural language processing, the practical effectiveness of adversarial debiasing in healthcare settings remains incompletely characterised. Clinical datasets often feature substantial class imbalance, small subgroup sizes for minority demographics, measurement error, and complex interactions (4) between demographic variables and clinical features—conditions that may challenge the assumptions underlying fairness algorithms developed on clean, balanced datasets. Moreover, fairness is inherently multidimensional: a model can improve equity by one metric while worsening it by another, and the optimal trade-off between predictive performance and fairness depends on the clinical context and stakeholder values (9). Recent studies have documented persistent algorithmic disparities across demographic groups in diverse healthcare settings (10–12), underscoring the need for evaluation frameworks that go beyond single-metric, single-split reporting.

Existing studies on fairness in clinical prediction have several limitations. Many rely on single train–test splits (6), which can produce misleading fairness assessments if the properties of subgroups vary across partitions. Few explicitly address the statistical challenges of fairness evaluation with small demographic subgroups (13), where sampling variability can dominate the observed disparities. Most studies focus on racial or ethnic disparities (14), with less attention to age-related bias despite the clinical importance of age-stratified care and the growing use of AI in geriatric medicine. Many reports demonstrate single-metric fairness improvements without exploring whether benefits persist across alternative fairness definitions.

This study addresses these gaps by evaluating adversarial debiasing for age-equitable diabetes prediction under realistic conditions of subgroup imbalance, with explicit attention to partition dependency and small-sample effects. We focus on age-related bias because, diabetes risk increases with age, but clinical presentation varies across age groups (15), creating potential for age-dependent model errors; older adults represent a small but high-risk population in many datasets, enabling the study of fairness interventions under subgroup imbalance; age can be discretised in multiple ways, providing a tractable setting for categorical fairness analysis; and age-related algorithmic bias has received less attention than racial bias despite its clinical significance.

The study makes five specific contributions. It provides an empirical evaluation of adversarial debiasing with a Gradient Reversal Layer (GRL) for age-equitable diabetes prediction, characterises performance–fairness trade-offs across subgroups, conducts robustness analysis across five independent random seeds to assess partition dependency, reports cases where debiasing improves, worsens, or has no effect on fairness, and presents an explicit analysis of how small subgroup sample sizes destabilise fairness metrics.

### 1.1 Research question and hypothesis

Research question: Does adversarial debiasing using a GRL improve age-equitable recall across three age groups (<30, 30–50, and >50 years) in a diabetes prediction model, compared to a logistic regression baseline, and are any observed fairness improvements robust to changes in data partition?

Hypothesis: Adversarial debiasing will reduce the recall parity gap across age groups compared to the baseline model. However, given the small size of the >50-year subgroup and the known sensitivity of fairness metrics to data partition, improvements may not be consistent across all random seeds.

## 2 Methods

### 2.1 Dataset and outcome definitions

This study used the Pima Indians Diabetes Database, a publicly available and fully de-identified dataset accessible via Kaggle (https://www.kaggle.com/datasets/uciml/pima-indians-diabetes-database; accessed December 21, 2025). The dataset originated from the National Institute of Diabetes and Digestive and Kidney Diseases (NIDDK) and contains diagnostic measurements from female patients of Pima Indian heritage, aged 21 years or older. No patient identifiers are present in the dataset. All 768 patients were included, and no additional exclusion criteria were applied beyond those inherent to the published dataset. The binary outcome variable was diabetes status (0 = non-diabetic, 1 = diabetic). The overall dataset included 500 non-diabetic (65.1%) and 268 diabetic (34.9%) patients. All eight predictor variables in the dataset were used as model inputs: number of pregnancies, plasma glucose concentration, diastolic blood pressure, triceps skinfold thickness, serum insulin, body mass index (BMI), diabetes pedigree function, and age. Age was used both as a predictive feature and to derive the categorical age group variable used for fairness evaluation; the derived age group variable was not included as a separate predictive feature. The age distribution across the full dataset was: <30 years (*n* = 417, 54.3%), 30–50 years (*n* = 270, 35.2%), and >50 years (*n* = 81, 10.5%).

The dataset contains zero values in five biological variables (glucose, blood pressure, BMI, insulin, and skin thickness) that are physiologically implausible and are commonly treated as missing values in the literature. In this analysis, these zero values were retained as recorded and used directly during model training and evaluation; no imputation or exclusion of zero values was performed. The implications of this choice are discussed in the Limitations. Predictor variables were standardised using a standard scaler (StandardScaler) fitted on the training data only; the fitted transformation was then applied to the test data. No information from the test set was used when fitting the scaler. No additional feature engineering beyond standardisation was performed. All analyses were conducted in TensorFlow 2.× (version 2.19.0) on a Python platform.

### 2.2 Age group stratification

Age was categorised into three groups: (1) <30 years, (2) 30–50 years, and (3) >50 years. This categorisation was chosen to capture distinct life stages associated with different diabetes risk profiles, including younger adults potentially affected by early-onset diabetes, including type 1 diabetes, middle-aged adults experiencing increasing metabolic risk, and older adults with cumulative exposure and higher complication rates (16).

Age serves a dual role in this analysis: it functions as both a protected attribute (for which fairness is assessed) and a clinically meaningful predictor of diabetes risk. Removing age-related information from the shared representation through adversarial training may therefore also reduce clinically relevant signal. The adversary accuracy of 0.6169 observed in this study indicates partial, but not complete, suppression of age information—which may represent a pragmatic compromise between fairness and clinical utility. Readers should interpret any observed fairness gains in the context of this trade-off.

### 2.3 Data splitting and seed strategy

The dataset was divided into training and testing sets using a split stratified by the diabetes outcome variable (Outcome) only; the age group variable was not used as a stratification variable. The primary results reported in the main tables correspond to a single labelled test partition of 154 patients (20.1% of the dataset). This partition is referred to here as the primary test partition. The manuscript originally labelled this partition as corresponding to a fixed random seed of 42; however, based on the analysis files currently available, this exact random state cannot be independently verified (see Limitations). The primary labelled results appear to correspond to one of the available partitions, but the precise random_state value cannot be confirmed from the available files. The test partition age distribution was: <30 years (*n* = 72, 46.8%), 30–50 years (*n* = 69, 44.8%), and >50 years (*n* = 13, 8.4%). Within this partition, 54 patients (35.1%) had diabetes and 100 (64.9%) were non-diabetic.

Because the train–test split was stratified by outcome only and not by age group, the small >50-year subgroup was not balanced across partitions, which contributes to the subgroup instability discussed in Section 5A and the Limitations.

To assess robustness, the complete training and evaluation pipeline was repeated across five independent random seeds (0, 1, 2, 3, 4), each generating a different train–test partition. Robustness metrics are reported as mean ± standard deviation (SD) across the five seeds. This multi-seed approach enables quantification of partition-dependent variability in both performance and fairness metrics, which is particularly important given the small size of the >50-year subgroup.

## 3 Model architectures

### 3.1 Baseline model: logistic regression

The baseline was a standard logistic regression classifier implemented as a single linear layer with a sigmoid activation function. This model predicts diabetes probability without incorporating fairness constraints and serves as the performance benchmark. It does not learn latent representations; all input features are used directly.

Note on architectural comparison: The baseline logistic regression model and the adversarial debiasing model differ in both architecture (linear vs. neural) and training objective (unconstrained vs. adversarially constrained). Observed performance differences may therefore reflect the effect of neural architecture as well as the debiasing procedure. A neural baseline model (trained without adversarial constraints but using the same shared-representation architecture) would enable a cleaner attribution of fairness effects to the GRL alone; however, such a model was not included in this study and represents a limitation of the current design.

### 3.2 Adversarial debiasing model

The adversarial debiasing model is designed to learn age-invariant representations for diabetes prediction. The architecture has four components. The first component is a dense layer with eight units and ReLU activation that transforms the input features into a compact intermediate representation. This representation is the common input to both the prediction head and the adversary. The second component is a dense layer with one unit and sigmoid activation, connected to the shared representation, that outputs diabetes probability (binary outcome). The third component is a dense layer with three units and softmax activation, connected to the shared representation via a GRL, that attempts to classify the patient's age group (three-class categorical outcome). The adversary is trained to predict age group; the GRL then prevents this information from propagating usefully to the shared representation. The fourth component is the Gradient Reversal Layer (GRL), which is inserted between the shared representation and the adversary head (7). During the forward pass, it acts as an identity function, passing values unchanged. During the backward pass, it reverses the sign of gradients by multiplying them by a negative constant (−*λ*). This reversal causes the shared representation to be updated in a direction that increases the adversary's prediction loss—in other words, the model actively learns to discard age-group information from the shared representation, while simultaneously minimising the diabetes prediction loss. The result is a representation that is informative for predicting diabetes but uninformative for predicting age group. The combined loss function is: L_total = L_outcome + *λ* × L_adversary, where L_outcome is the binary cross-entropy for diabetes prediction, L_adversary is the sparse categorical cross-entropy for age group prediction, and *λ* is the adversarial strength hyperparameter controlling the relative weight of the debiasing objective.

## 4 Training configuration

All models were trained using the Adam optimizer with a learning rate of 0.001, a batch size of 32, and 40 epochs. The adversarial strength hyperparameter *λ* was selected by grid search over the values {0.0, 0.1, 0.2, 0.3, 0.4}. A separate validation partition was not used; *λ* was selected using model performance (recall) evaluated on the test partition. This constitutes exploratory, test-partition-based hyperparameter selection and introduces test-set leakage, because the same partition informed both hyperparameter selection and the reported evaluation. This limitation is disclosed in the Methods, Discussion, Limitations, and Conclusion. The final model used *λ* = 0.2.

**Figure 1:**
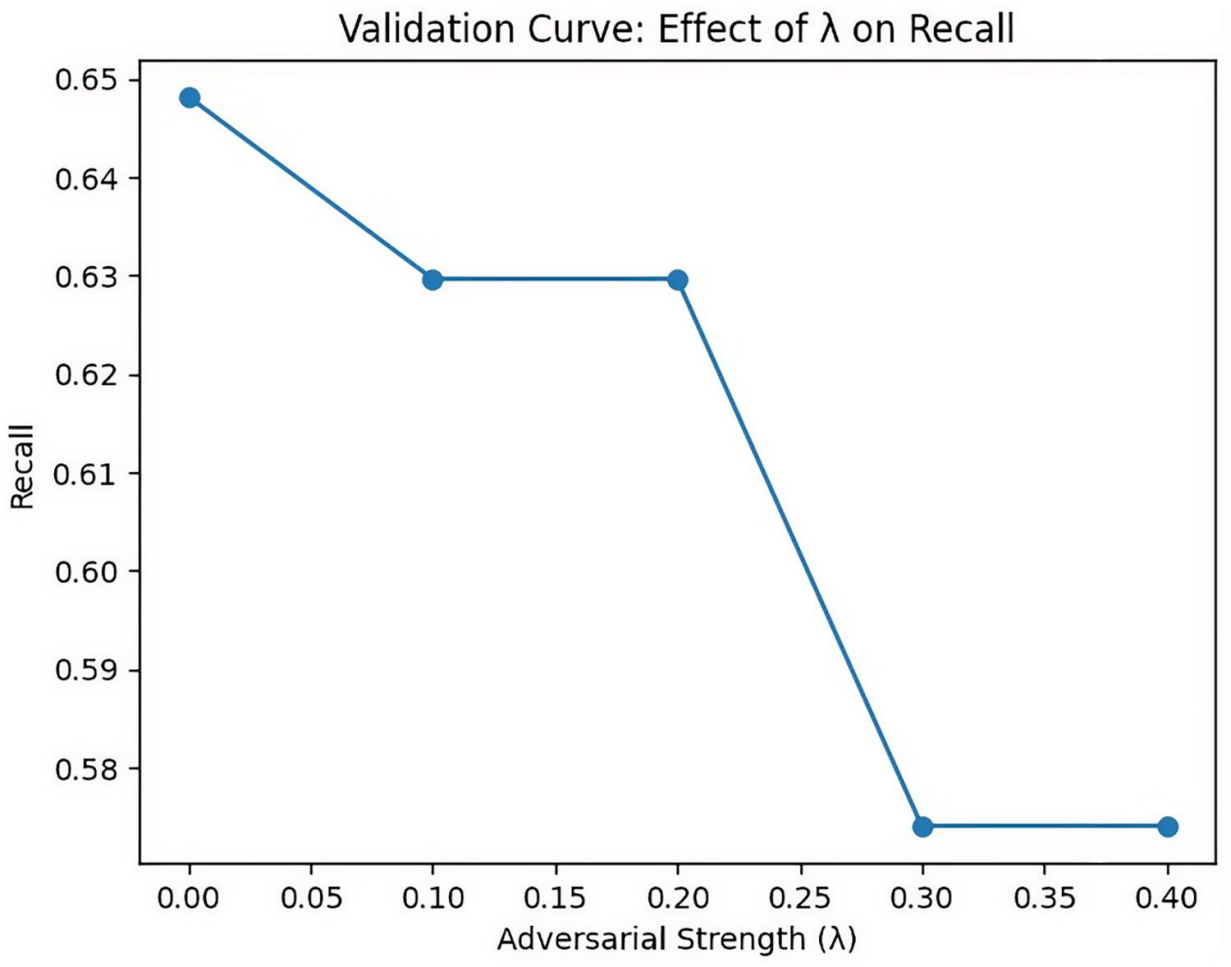
Tuning curve showing model recall on the test partition as a function of the adversarial strength parameter *λ*. Recall declines beyond *λ* = 0.2; *λ* = 0.2 was selected as the point at which moderate age-information suppression was achieved without substantial recall loss. *λ* was selected using the test partition rather than an independent validation partition (see Methods and Limitations).

As shown in Figure 1, recall on the test partition was highest at *λ* = 0.0 (0.648) and declined with increasing adversarial strength, reaching a plateau near 0.577 at *λ* ≥ 0.3. The selected value *λ* = 0.2 represents a point at which recall remained acceptable (0.630) while a moderate degree of age-information suppression was achieved (adversary accuracy: 0.6169). Increasing *λ* above 0.2 produced notable degradation in test-partition recall. Two limitations of this selection procedure should be emphasised: first, selection was based on recall rather than a fairness objective; and second, selection used the same partition that was used for the reported evaluation, introducing test-set leakage. Hyperparameter selection on an independent validation partition would be expected to yield less optimistic and more generalisable estimates.

All implementations were conducted in TensorFlow 2.× (version 2.19.0) (Figure 1).

## 5 Evaluation metrics

### 5.1 Overall performance metrics

Model performance was assessed using: accuracy (proportion of correct predictions); precision (TP/[TP + FP]); recall/sensitivity (TP/[TP + FN]); F1 score (harmonic mean of precision and recall); specificity (TN/[TN + FP]); ROC-AUC (area under the receiver operating characteristic curve) (17); and confusion matrix cell counts (TP, FP, TN, FN).

### 5.2 Group-wise performance metrics

To assess fairness, the following metrics were computed separately for each age group: TP, FP, TN, and FN counts; recall (TPR = TP/[TP + FN]); specificity (TNR = TN/[TN + FP]); and false positive rate (FPR = FP/[FP + TN]).

### 5.3 Fairness metric

Fairness was quantified using the recall parity gap, defined as Recall parity gap = max (Recall) − min (Recall) across age groups. Lower values indicate more equitable sensitivity across groups. This metric is particularly relevant for clinical screening applications, where missing a true case (false negative) carries direct patient-level consequences (18).

Given that the >50-year subgroup contained only 9 diabetic cases in the primary test partition, each correctly or incorrectly classified patient changes group recall by approximately 11 percentage points. Observed recall and recall parity gap values for this group are therefore highly sensitive to single-patient classification changes and should be interpreted with this instability in mind.

### 5.4 Robustness assessment

Stability was evaluated by repeating the full pipeline across five independent random seeds (0–4). For each seed, separate train–test splits were generated and both models were trained and evaluated. Robustness was summarised as mean ± SD across the five seeds.

### 5.5 Statistical and implementation details

All numerical results are reported to the precision present in the source data files. No additional statistical significance tests were conducted; results are based on descriptive comparison of metrics. Model training, evaluation, and fairness analysis were implemented using TensorFlow 2.19.0 on Python.

#### 5.5.1 Technical note on partition dependency and small-subgroup instability

Fairness metrics computed from a single train–test split may be unreliable, particularly when minority subgroups are small. This is not merely a limitation of study design; it is a fundamental statistical issue with important practical implications for how fairness evaluations of clinical ML models are interpreted and reported.

In this study, the >50-year age group comprised 13 patients in the primary test partition, of whom only 9 had diabetes. Given that recall is computed as TP/(TP + FN), each change of one correctly classified diabetic patient in this subgroup shifts recall by 1/9 ≈ 11.1 percentage points. Two additional correctly classified patients thus yield a 22.2 pp improvement—precisely the magnitude observed when comparing the baseline to the adversarial model in the primary test partition. Such a large percentage-point change may therefore reflect the reclassification of one or two individuals rather than a systematic shift in model behaviour.

This instability propagates to the recall parity gap because the gap is computed from the maximum and minimum group-level recalls. When the recall of a small subgroup changes by a large absolute amount due to single-patient reclassification, the parity gap changes accordingly, irrespective of whether the underlying model representation has changed meaningfully. The result is high variance in the parity gap across data partitions, as confirmed by the SD > 0.27 observed across seeds in this study.

Multi-seed evaluation does not eliminate this instability; it quantifies it. The key implication for practitioners is that single-split fairness evaluations with small subgroups should be treated as preliminary estimates rather than definitive assessments. Reporting the SD of the parity gap alongside its mean, as done in this study, provides a more honest characterisation of the reliability of fairness conclusions.

## 6 Results

### 6.1 Test partition composition (primary test partition)

The primary test partition comprised 154 patients: <30 years (*n* = 72, 46.8%), 30–50 years (*n* = 69, 44.8%), and >50 years (*n* = 13, 8.4%). Diabetic case counts within each group were: <30 years (16 diabetic, 56 non-diabetic), 30–50 years (29 diabetic, 40 non-diabetic), and >50 years (9 diabetic, 4 non-diabetic). The >50-year group was the smallest subgroup, with only 9 diabetic cases. The clinical implications of this are described in Section 5A.

### 6.2 Overall performance (primary test partition)

**Figure 2:**
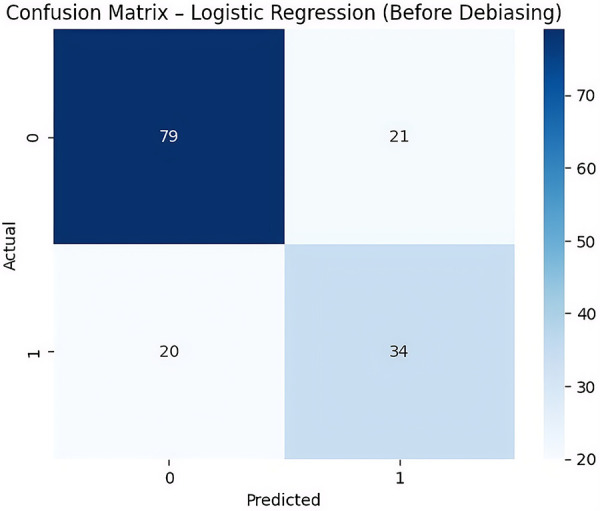
Confusion matrix for the baseline logistic regression model on the primary test partition (*n* = 154). The model achieved 34 true positives, 79 true negatives, 21 false positives, and 20 false negatives, corresponding to an accuracy of 0.7338 and a recall of 0.6296.

**Figure 3:**
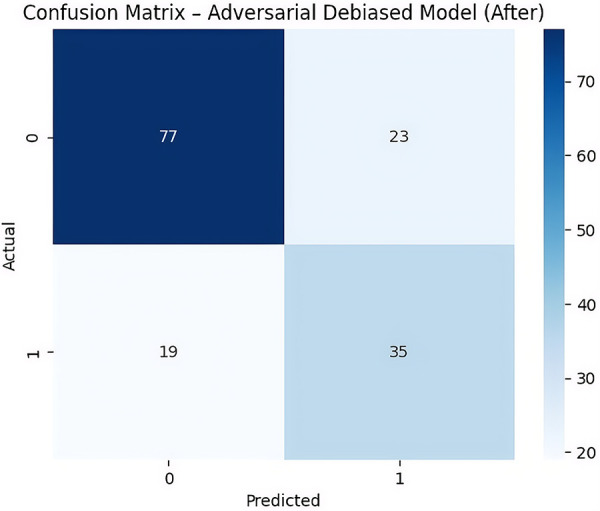
Confusion matrix for the adversarial debiasing model on the primary test partition (*n* = 154). The model achieved 35 true positives, 77 true negatives, 23 false positives, and 19 false negatives, corresponding to an accuracy of 0.7273 and a recall of 0.6481.

**Figure 4:**
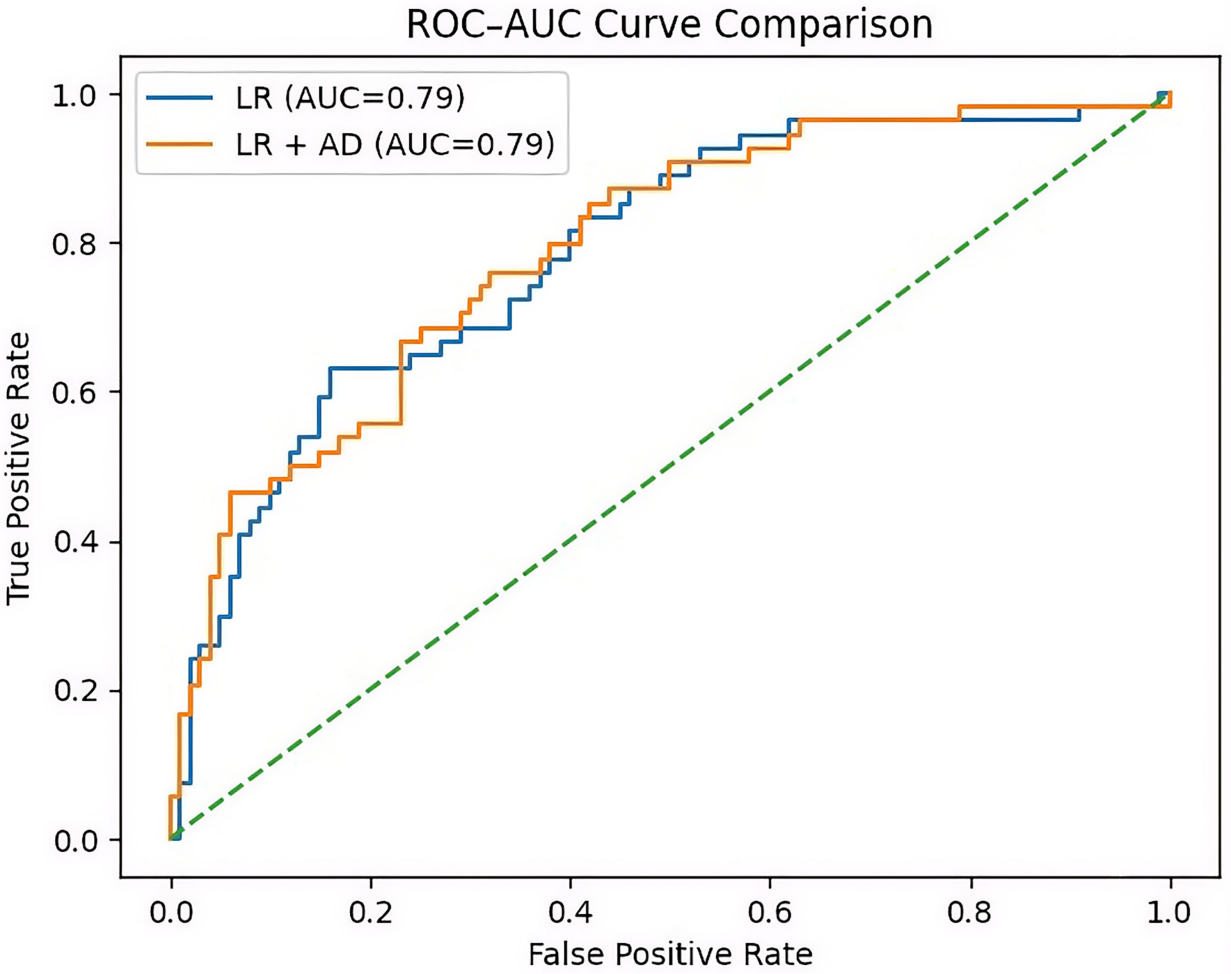
Receiver operating characteristic (ROC) curves for the baseline logistic regression and adversarial debiasing models on the primary test partition (*n* = 154). The adversarial model achieved a marginally higher ROC-AUC (0.7896) than the baseline model (0.7852), indicating comparable overall discriminative performance.

Table 1 presents overall classification performance metrics for both models on the primary test partition (*n* = 154). The adversarial model showed a small improvement in recall (+1.85 pp) and ROC-AUC (+0.45 pp) relative to the baseline, with modest decreases in accuracy (−0.65 pp), precision (−1.47 pp), and specificity (−2.00 pp). Confusion matrix analysis: the adversarial model correctly identified one additional diabetic case (TP: 34 → 35, FN: 20 → 19) at the cost of two additional false positives (FP: 21 → 23, TN: 79 → 77). The overall change in discriminative performance was marginal (Figures 2–4).

**Table 1:** Overall performance metrics for the baseline logistic regression and adversarial debiasing models on the primary test partition (*n* = 154). TP: true positives; FP: false positives; TN: true negatives; FN: false negatives; pp: percentage points.

Model	Accuracy	Precision	Recall	F1	Specificity	ROC-AUC	TP	FP	TN	FN
Baseline (LR)	0.7338	0.6182	0.6296	0.6239	0.7900	0.7852	34	21	79	20
Adversarial	0.7273	0.6034	0.6481	0.6250	0.7700	0.7896	35	23	77	19
Change	−0.65 pp	−1.47 pp	+1.85 pp	+0.11 pp	−2.00 pp	+0.45 pp	+1	+2	−2	−1

LR, logistic regression. Change row shows adversarial minus baseline.

### 6.3 Age-stratified performance and fairness analysis (primary test partition)

#### 6.3.1 Baseline model

Table 2 presents the age-stratified performance for the baseline logistic regression model. Recall was highest in the 30–50-year group (0.6552; 19 of 29 diabetic cases correctly identified) and lowest in the >50-year group (0.5556; 5 of 9 correctly identified). Specificity was notably low for the >50-year group (0.2500), indicating that only 1 of 4 non-diabetic patients in this subgroup was correctly identified as non-diabetic. The baseline recall parity gap was 0.0996 [max: 0.6552 (30–50 years), min: 0.5556 (>50 years)].

**Table 2:** Age-stratified performance metrics for the baseline logistic regression model (primary test partition).

Age group	TP	FP	TN	FN	Recall (TPR)	Specificity (TNR)	FPR
<30 years	10	9	47	6	0.6250	0.8393	0.1607
30–50 years	19	9	31	10	0.6552	0.7750	0.2250
>50 years	5	3	1	4	0.5556	0.2500	0.7500
Recall Parity Gap	—	—	—	—	0.0996	—	—

TPR, true positive rate; TNR, true negative rate; FPR, false positive rate. Recall parity gap = max(Recall) − min(Recall) across groups.

#### 6.3.2 Adversarial debiasing model

Table 3 presents the age-stratified performance for the adversarial debiasing model. Key findings are as follows. The >50-year group showed a recall improvement from 0.5556 to 0.7778 (+22.22 pp), equivalent to two additional diabetic cases correctly identified (5 → 7 of 9). However, as discussed in Section 5A, each change of one correctly classified patient in this group represents ≈11.1 pp of recall; the observed improvement reflects the reclassification of two individuals and should not be interpreted as a stable, large-magnitude effect. The <30-year group recall declined from 0.6250 to 0.5625 (−6.25 pp; 10 → 9 of 16 correctly identified). The 30–50-year group recall was unchanged at 0.6552. The adversarial recall parity gap increased to 0.2153 [max: 0.7778 (>50 years), min: 0.5625 (<30 years)]—an increase of 11.57 pp compared to the baseline. This apparent paradox is explained in Section 7.

**Table 3:** Age-stratified performance metrics for the adversarial debiasing model (primary test partition).

Age group	TP	FP	TN	FN	Recall (TPR)	Specificity (TNR)	FPR
<30 years	9	9	47	7	0.5625	0.8393	0.1607
30–50 years	19	12	28	10	0.6552	0.7000	0.3000
>50 years	7	2	2	2	0.7778	0.5000	0.5000
Recall Parity Gap	—	—	—	—	0.2153	—	—

TPR, true positive rate; TNR, true negative rate; FPR, false positive rate. Recall parity gap = max(Recall) − min(Recall) across groups.

Interpretation note for Table 3: The adversarial model increased recall for the >50-year group by 22.22 pp. Because this group contained only 9 diabetic patients, this change corresponds to 2 additional correctly classified individuals. Simultaneously, recall for the <30-year group fell by 6.25 pp (1 fewer correctly classified patient among 16 diabetic cases). The increase in the recall parity gap (0.0996 → 0.2153) is therefore driven by these small absolute changes at the patient level rather than by a systematic distributional shift. The specificity of the >50-year group improved from 0.2500 to 0.5000, indicating that 1 additional non-diabetic patient in this subgroup was correctly classified as non-diabetic.

### 6.4 Multi-seed robustness: overall performance (seeds 0–4)

Table 4 presents mean ± SD of overall performance metrics across five random seeds. The adversarial model showed higher variability in recall (SD: 0.0930 vs. 0.0685 for baseline) and F1 score (SD: 0.0668 vs. 0.0476), indicating that the adversarial training introduced additional instability in overall performance. Mean accuracy, precision, and ROC-AUC were similar between models.

**Table 4:** Mean ± SD of overall performance metrics for baseline and adversarial models across five random seeds (0–4).

Metric	Baseline (Mean ± SD)	Adversarial (Mean ± SD)
Accuracy	0.7558 ± 0.0282	0.7494 ± 0.0397
Precision	0.7013 ± 0.0673	0.6793 ± 0.0686
Recall	0.5407 ± 0.0685	0.5519 ± 0.0930
F1 Score	0.6073 ± 0.0476	0.6049 ± 0.0668
Specificity	0.8720 ± 0.0492	0.8560 ± 0.0550
ROC-AUC	0.8162 ± 0.0395	0.8089 ± 0.0306

### 6.5 Multi-seed robustness: subgroup recall and parity gap (seeds 0–4)

**Figure 5:**
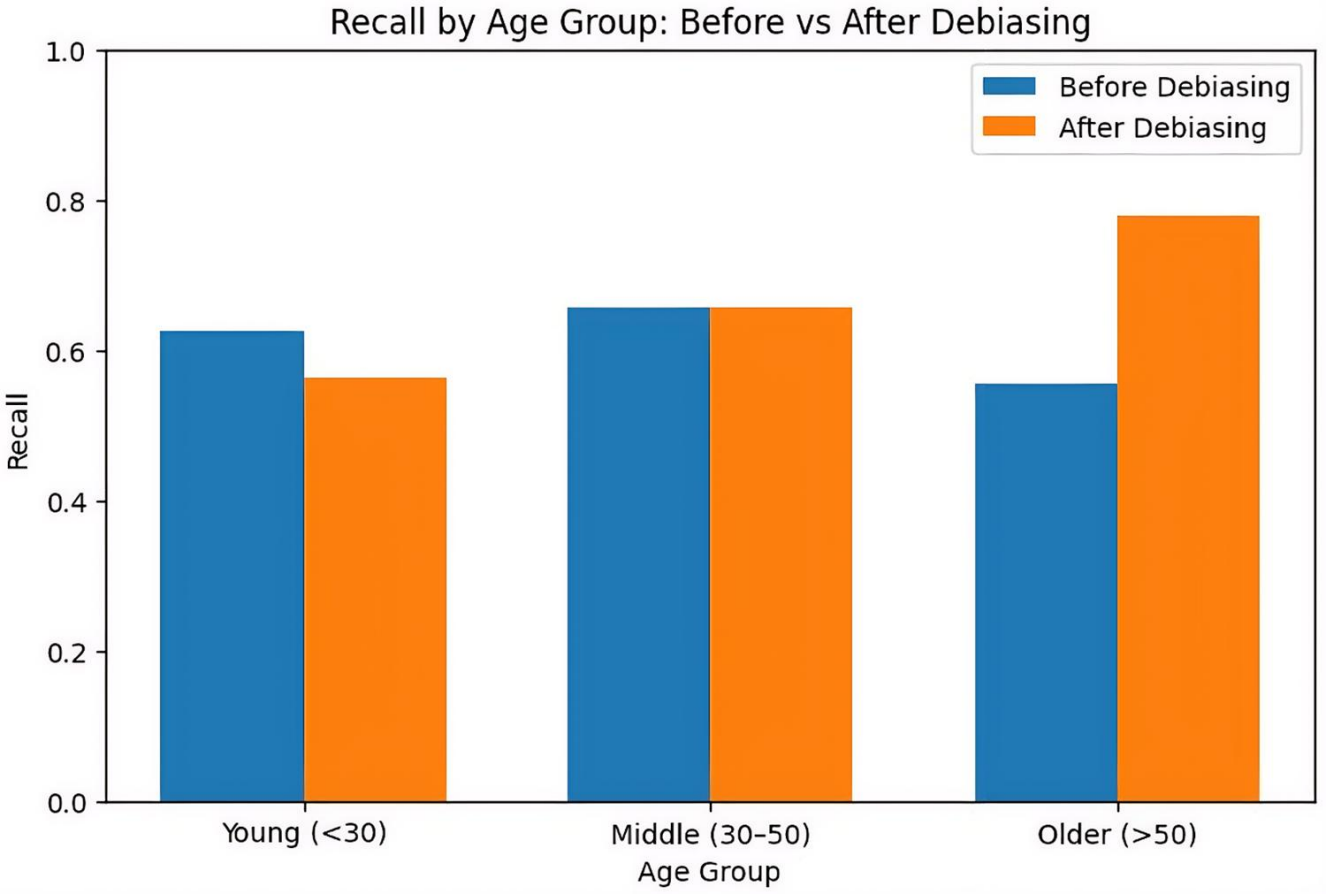
Comparison of subgroup recall across age groups (<30 years, 30–50 years, and >50 years) for the baseline logistic regression and adversarial debiasing models, averaged across five random seeds (0–4). The adversarial model modestly improved mean recall for the 30–50-year group but showed high variability across seeds, particularly for the >50-year group (SD > 0.20), illustrating the partition-dependent nature of fairness outcomes.

Table 5 presents age-group-specific recall and the recall parity gap across five seeds. The >50-year group exhibited the highest SD in recall for both models (baseline: 0.1860; adversarial: 0.2011), reflecting high sensitivity to small-sample composition. Individual-seed recall for this group ranged from 0.5556 to 1.0000 (baseline) and 0.5000 to 1.0000 (adversarial), illustrating extreme partition dependence. The mean recall parity gap improved modestly with adversarial debiasing (0.3282 → 0.3033, −2.49 pp), but the SD (>0.27) exceeded the mean difference. The adversarial model reduced the parity gap in 3 of 5 seeds, increased it in 1, and produced no change in 1 (Figure 5).

**Table 5:** Mean ± SD of age-group recall and recall parity gap across five random seeds (0–4).

Metric	Baseline (Mean ± SD)	Adversarial (Mean ± SD)
Recall (<30 years)	0.4501 ± 0.1410	0.4397 ± 0.1293
Recall (30–50 years)	0.5721 ± 0.0680	0.5987 ± 0.1064
Recall (>50 years)	0.7444 ± 0.1860	0.7333 ± 0.2011
Recall Parity Gap	0.3282 ± 0.2811	0.3033 ± 0.2710

## 7 Performance–fairness trade-off summary

The adversarial debiasing approach demonstrated complex, partition-dependent performance–fairness trade-offs. On the primary test partition, the model improved recall for the >50-year group (+22.22 pp) while maintaining comparable overall performance, but the recall parity gap increased by 11.57 pp. This counterintuitive result—improving the performance of the worst-performing group while widening overall disparity—arises because the <30-year group recall declined simultaneously. The recall parity gap reflects the worst-case difference across all groups; when the minimum-recall group improves but another group deteriorates, the gap can increase even if the previously disadvantaged group benefits.

Across five seeds, the mean parity gap showed a modest mean reduction (−2.49 pp) with high variability (SD > 0.27) and heterogeneous effects: improved in 3 of 5 seeds, worsened in 1, and unchanged in 1. These results collectively indicate that adversarial debiasing can selectively enhance recall for underrepresented subgroups, but does not guarantee consistent or clinically meaningful fairness improvements when subgroup sizes are small and data partitions vary.

## 8 Discussion

This study evaluated adversarial debiasing with a gradient reversal layer as a method for mitigating age-related bias in diabetes prediction. The findings demonstrate a complex, partition-dependent performance–fairness trade-off that challenges simplified accounts of algorithmic fairness interventions. Although adversarial debiasing substantially improved recall for the smallest and previously worst-performing age group (>50 years) on the primary test partition, this improvement was accompanied by an increase in overall recall disparity across age groups. Across multiple random seeds, the method showed a modest mean fairness improvement with high variability, underscoring the fundamental difficulty of achieving consistent equitable performance when working with imbalanced subgroup sizes.

### 8.1 Improved performance for underrepresented subgroups

On the primary test partition, the adversarial model improved recall for the >50-year group from 0.5556 to 0.7778 (+22.22 pp), corresponding to two additional correctly identified diabetic cases from nine total. This addresses a clinically important concern: older adults with diabetes face higher complication rates (19) and often present with atypical symptoms that complicate diagnosis (20). The improvement occurred while overall performance remained comparable, suggesting a partial reallocation of predictive capacity toward the previously underserved age group. Similar findings have been reported in healthcare machine learning studies using adversarial training frameworks, where bias mitigation was achieved while maintaining clinically useful predictive performance (21). An adversary accuracy of 0.6169 indicates partial, but not complete, suppression of age-related information from the shared representation, which may represent an appropriate balance between fairness and clinical utility.

### 8.2 The fairness paradox: parity gap increase on primary split

Despite improved recall for the >50-year group, the adversarial model increased the recall parity gap from 0.0996 to 0.2153. This paradox arises from concurrent changes across multiple subgroups: while the >50-year group improved (+22.22 pp), the <30-year group declined (−6.25 pp), widening the overall disparity. This illustrates a fundamental principle: optimising one fairness criterion—here, improving the worst-performing group—does not guarantee improvement in aggregate fairness metrics. The recall parity gap captures the worst-case disparity between any two groups; when the originally worst-performing group improves but another group deteriorates, the gap reflects the new worst-case difference rather than the historical one.

This finding aligns with recent work documenting the multidimensionality of algorithmic fairness in healthcare (10, 22). Bias mitigation strategies optimised for one subgroup or one metric may produce unintended redistribution of prediction errors across other subgroups. Similar fairness–accuracy trade-offs have been documented in clinical risk prediction models, where improvements in subgroup equity may be accompanied by changes in overall predictive performance (23). Abakasanga et al. (12) similarly observed in a hospital length-of-stay prediction study that bias mitigation improved fairness for some demographic subgroups while producing differential effects across others, even when overall model performance was maintained.

### 8.3 Robustness analysis: partition-dependency and small-sample effects

The robustness analysis revealed substantial variability, particularly in the >50-year age group (SD: 0.2011 for adversarial recall). With only 9–13 diabetic patients in this subgroup across different test sets (24), a change of one or two correctly classified cases produces large percentage-point swings in recall. Across five seeds, the mean parity gap improved modestly (−2.49 pp), but the SD exceeded the mean difference, indicating that the observed improvement is not statistically distinguishable from sampling variability. The adversarial model reduced the gap in 3 of 5 seeds, increased it in 1, and produced no change in 1, underscoring the partition-dependent nature of fairness outcomes.

These results are consistent with the broader literature on model evaluation with small demographic subgroups (13). Atehortúa et al. (11) demonstrated in a cardiometabolic risk prediction study that fairness evaluations across demographic subgroups require adequate subgroup sample sizes for reliable assessment; their multi-centre validation approach provides a useful model for future work in this domain.

### 8.4 Implications for fairness-aware machine learning in healthcare

The findings of this study highlight several important considerations for fairness-aware machine learning in diabetes prediction. Fairness is multidimensional and metric-dependent (22, 25), and improving one fairness metric does not guarantee improvements in others. Small subgroup sizes fundamentally limit the precision of fairness measurement and optimisation. In this dataset, the >50-year group is too small for stable fairness conclusions from any single split. Single-split evaluations are insufficient, and multi-seed evaluation is essential to distinguish stable effects from sampling artefacts (26). Adversarial debiasing is not a universal solution, and its effectiveness depends critically on dataset characteristics, particularly subgroup size and class balance (8). Fairness interventions may inadvertently target sampling noise rather than systematic model bias when subgroups are small. Furthermore, the architectural difference between the baseline (logistic regression) and the adversarial model (neural network with shared representation) means that observed performance differences may reflect model complexity rather than the debiasing procedure alone. Finally, because *λ* was selected on the test partition rather than an independent validation partition, the reported adversarial results are subject to test-set leakage and should be regarded as exploratory. An unbiased evaluation would require hyperparameter selection on data held out from the final test partition.

## 9 Limitations

Several limitations should be considered when interpreting these findings. The >50-year subgroup comprised only 13 patients (8.4%) in the primary test partition, including 9 diabetic cases that form the recall denominator. Because recall for this group changes by approximately 11.1 percentage points per patient reclassification, the observed recall and parity gap values are highly sensitive to the composition of individual test partitions. The high SD (>0.20) across seeds directly quantifies this limitation, and larger and more balanced datasets are required for reliable fairness assessment. Fairness was quantified using the recall parity gap, which emphasises worst-case disparities, although alternative metrics such as demographic parity, equalised odds, and calibration difference might yield different conclusions. Metric choice should be guided by stakeholder values, which were not systematically elicited in this study. All analyses used the Pima Indians Diabetes Database, which contains only female patients of Pima Indian heritage aged ≥21 years, and external validation in independent, geographically and ethnically diverse cohorts is necessary before conclusions can be generalised. The baseline model (logistic regression) and the adversarial model (neural network) differ in both architecture and training objective, and a neural baseline without adversarial constraints was not evaluated; therefore, observed differences in performance and fairness may reflect architectural differences rather than the effect of debiasing alone. The use of three discrete age groups simplifies continuous age, potentially obscuring within-group heterogeneity and creating artificial boundary effects. In addition, only baseline logistic regression was compared, whereas reweighting, threshold optimisation, and ensemble methods may achieve different performance–fairness trade-offs. Zero values in glucose, blood pressure, BMI, insulin, and skin thickness were retained as recorded and used directly in model training and evaluation without imputation or exclusion. Some of these zero values are biologically implausible and likely represent missing data, and retaining them may have introduced noise and affected both overall and subgroup-level performance, thereby limiting reproducibility and comparability with studies that impute or exclude such values. Furthermore, machine learning metrics were evaluated rather than clinical outcomes such as time to diagnosis or complication rates (27). The adversarial strength hyperparameter *λ* was selected using performance on the same test partition used for the reported evaluation rather than on an independent validation partition, introducing test-set leakage and likely producing optimistic performance and fairness estimates for the adversarial model. The reported metrics should therefore be interpreted as exploratory rather than as unbiased estimates of generalisation performance. In addition, *λ* was selected using recall rather than a fairness objective, and strategies that directly optimise the parity gap on an independent partition may yield different results. The manuscript originally labelled the primary analysis as corresponding to a fixed random seed (random_state = 42); however, based on the analysis files currently available, this exact random state cannot be independently verified. The primary labelled results appear to correspond to one of the available partitions, but the precise seed cannot be confirmed without recovering the original analysis files. Although robustness analyses were conducted across five independent random seeds (0–4), the train–test split was stratified only by diabetes outcome and not by age group. As a result, the small >50-year subgroup was not balanced across partitions, contributing to the partition-dependent instability of subgroup recall and the recall parity gap. Finally, the binary outcome and single time point do not capture diabetes heterogeneity, including disease type, severity, or progression; fairness assessment was limited to age and did not consider intersections with sex, race, or socioeconomic status (28); and the original data collection context is unknown, making the generalisability of these findings to contemporary or geographically distinct populations uncertain.

## 10 Conclusions

Adversarial debiasing with a gradient reversal layer improved diabetes prediction recall for older adults (>50 years, +22.22 percentage points on the primary test partition), but produced inconsistent fairness outcomes across different data partitions. On the primary test partition, the recall parity gap increased from 0.0996 to 0.2153 despite improved recall for the >50-year group, because recall for the <30-year group declined simultaneously. Across five random seeds, the mean parity gap showed only a modest reduction (0.3282 → 0.3033, −2.49 pp) with a standard deviation exceeding the mean difference, indicating that observed improvements cannot be distinguished from sampling variability.

These findings do not support the conclusion that adversarial debiasing has solved the fairness problem in this setting. They do indicate that the method can selectively improve recall for underrepresented subgroups, but that this improvement is unstable and may be accompanied by fairness degradation in other subgroups when evaluated using the recall parity gap. Small subgroup sample sizes (13 patients in the >50-year subgroup, including 9 diabetic cases that form the recall denominator) amplify this instability by making subgroup-level metrics highly sensitive to individual patient classifications. Two further caveats constrain these conclusions: the adversarial strength hyperparameter *λ* was selected on the test partition rather than an independent validation partition, introducing test-set leakage that makes the reported adversarial results exploratory rather than unbiased; and the exact random state of the primary labelled partition could not be independently verified from the available analysis files.

Achieving equitable clinical machine learning requires: multi-seed evaluation rather than single-split reporting; fairness metric selection aligned with stakeholder values and clinical context; larger, more balanced, and more demographically diverse datasets for reliable fairness assessment; and explicit acknowledgement that no single algorithmic intervention guarantees consistent fairness improvements across all data partitions and evaluation metrics.

### 10.1 Future directions

Several directions for future research emerge from this work. First, larger datasets with adequate representation of older adults are needed to enable stable fairness measurement; prospective collection of age-balanced diabetes cohorts would substantially improve the precision of parity gap estimates. Second, direct comparison with other in-processing fairness methods (reweighting, fairness constraints as regularisation, equalized odds optimisation) and postprocessing methods (threshold calibration by group) would enable a more comprehensive understanding of performance–fairness trade-offs. Third, including a neural baseline (same shared-representation architecture, without adversarial constraints) would disentangle the effects of model complexity from the effects of the GRL. Fourth, extending the evaluation to intersectional subgroups (defined jointly by age, sex, and ethnicity) would provide a more complete picture of how debiasing affects multiple overlapping demographic categories (28). Fifth, prospective clinical validation—measuring not only ML metrics but also clinical outcomes such as time to diagnosis and complication rates—is needed before adversarial debiasing approaches can be considered for deployment in clinical decision support systems. Finally, future studies should consider whether directly optimising a fairness objective (rather than overall recall) during *λ* selection leads to more reliable fairness improvements across partitions.

## Data Availability

Publicly available datasets were analyzed in this study. This data can be found here: https://www.kaggle.com/datasets/uciml/pima-indians-diabetes-database.
